# Interim guidance for health‐care professionals and administrators providing hospital care to adult patients with cognitive impairment, in the context of COVID‐19 pandemic

**DOI:** 10.1111/ajag.12831

**Published:** 2020-10-13

**Authors:** Melinda Martin‐Khan, Kasia Bail, Mark W. Yates, Jane Thompson, Fred Graham, Cognitive Impairment and COVID‐19, Hospital Care Guidance Committee, Melinda Martin‐Khan, Alison Argo, Kasia Bail, Gideon Caplan, Denise Craig, Anne Cumming, Stephanie Ellis, Leon Flicker, Amanda Fox, Dennis Frost, Jennifer Galstuch‐Leon, Frederick Graham, AnnMarie Hosie, Juanita Hughes, Leanne Jack, David Lie, R. J. Soares Magalhães, Elizabeth Miller, Glenys Petrie, Ranjeev Chrysanth Pulle, John Quinn, Bobby Redman, Linda Schnitker, Christine Stirling, Eddy Strivens, Mark Yates

**Affiliations:** ^1^ Faculty of Medicine Centre for Health Services Research University of Queensland Woolloongabba Qld Australia; ^2^ University of Canberra – Nursing Bruce, Canberra ACT Australia; ^3^ School of Medicine Deakin University Faculty of Health Medicine Nursing and Behavioural Sciences Burwood Vic. Australia; ^4^ Faculty of Medicine Centre for Health Services Research eQC Patient and Carer Advisory Board University of Queensland Woolloongabba Qld Australia; ^5^ Queensland Health Princess Alexandra Hospital Brisbane Qld Australia; ^6^ Faculty of Medicine Centre for Health Services Research The University of Queensland Herston Qld Australia

**Keywords:** cognitive impairment, COVID‐19, Hospital, physical and pharmacological restraints, delirium

## Abstract

**Objective:**

We developed interim guidance for the care of patients with cognitive impairment in hospital during the COVID‐19 pandemic.

**Methods:**

A Guidance Committee and Readers Group were recruited. The content was identified by the Committee and content‐specific subgroups, resulting in a draft document, which was sent to the Readers for review. People with dementia and care partners were involved in all aspects of the process.

**Results:**

Infection control measures can lead to an escalation of distress. In an environment where visiting bans are applied to care partners/advocates, hospitals need to ensure care partners can continue to provide decision‐making support. Health‐care professionals can proactively engage care partners using videoconferencing technologies. Developing models of care that proactively support best practice can minimise the risk of delirium, mitigate escalating symptoms and guide the use of non‐pharmacological, pharmacological (start low, go slow) or physical restraint in managing behavioural and psychological symptoms.

## 1 PATIENTS WITH COGNITIVE IMPAIRMENT IN HOSPITAL DURING COVID‐19 PANDEMIC

Interim guidance for health‐care professionals and administrators providing hospital care to adult patients with cognitive impairment, in the context of COVID‐19 pandemic. More information at https://chsr.centre.uq.edu.au/interim-guidance-care-adult-patients-cognitive-impairment-requiring-hospital-care-during-covid-19-pandemic-australia

## 2 COGNITIVE IMPAIRMENT MAY INCREASE DURING COVID‐19

- COVID‐19 can cause delirium
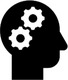

- Admissions may increase for patients with dementia or intellectual disability due to COVID‐19 spatial isolation and reduced community resources
- Patients with any kind of cognitive impairment are at higher risk of complications and distress, for example adverse events, long length of stay, behavioural and psychological symptoms and death
- Higher risk warrants increased preventative strategies to reduce the risk of harm

## 3 PEOPLE WITH COGNITIVE IMPAIRMENT MAY REQUIRE INNOVATIVE APPROACHES TO CARE BECAUSE OF:

- Inconsistent historians, comprehension of care requirements and remembering/following instructions
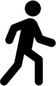

- Challenges in maintaining infection control principles (eg keeping mask on) due to the person experiencing anxiety, restlessness, breathlessness, exit‐seeking behaviours/wandering, fear, agitation or aggression
- Limited access to their usual care partner/advocate (eg due to COVID‐19 control measures or illness)
- Fear of people wearing PPE, which can be frightening and unfamiliar

## 4 CLINICAL STRATEGIES TO MAINTAIN EFFICIENT, EFFECTIVE AND ETHICAL CARE

**Figure 1:**
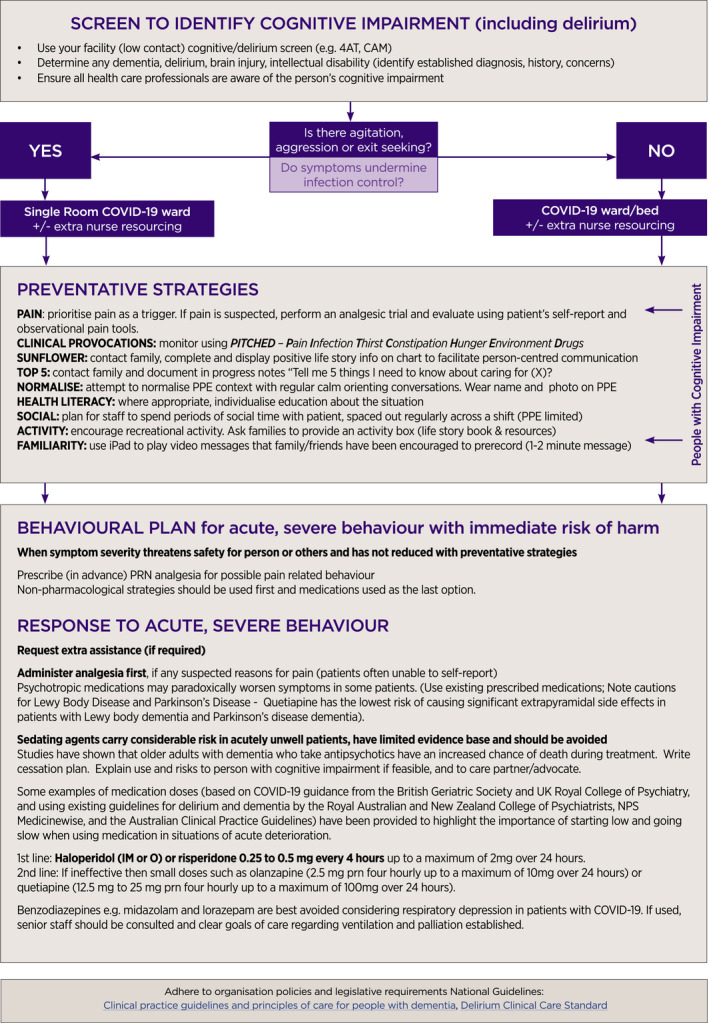
Flow chart of COVID‐19–related care decisions for people with cognitive impairment. The full 2‐page version of this poster can be viewed in the article’s Online Supporting Information.

- Assess patients to identify contributing factors to delirium and factors that are treatable
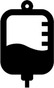

  - Manage hypoxia, pain, infection, dehydration, constipation, hunger and strange environments
  - Reduce polypharmacy and tethers where possible (IVC, IDC and bed rails)
- Normalise infection control practices
  - Use regular calm reorienting conversations, maintain calm demeanour, prioritise dignity and respect
  - Provide sample packs of personal protective equipment (PPE) to enable patients’ familiarisation
  - Consider humanisation of staff by placing large print name labels and photographs on staff wearing PPE
  - Consider the best environment for individual patients based on their acceptance of PPE
  - Provide education on PPE and infection control to care partner/advocate who will be present in hospital
- Orient people with cognitive impairment using biopsychosocial reinforcement
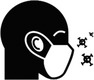

  - Welcome care partner/advocates to stay with people with cognitive impairment
  - Document the ‘Top 5’ strategies that were requested by the person (or care partner/advocate) for help with their care in their medical record
  - Place items in view (family photographs, music, phone and personal items)
  - Encourage activity (life storybook/app, puzzles, fidget boards, towel folding and toolbox)
  - Use human solutions (hearing and visual aids, music, pictures, TV and video)
  - Support time orientation: day/night lighting; bedside clock/calendar; and assist with meals
  - Promote the use of staff familiar to the patient; social and mobilising time
  - Write down information and instructions for patients, use visible whiteboard
- Discuss and document goals of care
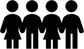

  - Identify the lawful decision‐maker if substitute decision‐making is occurring
  - Support shared decision‐making, informed consent and advance care planning
  - Plan comprehensive care based on the patient’s goals of care, and in line with their values and preferences, ensure regular communication
  - Focus on reablement, palliative care or end‐of‐life care as relevant
- Respond to any behavioural crisis (breach of infection control and aggressive behaviour) (Figure 1)
  - Implement non‐pharmacological strategies (as above)
  - Medications should be avoided and used only in extreme circumstances in a timely manner with consent policies and procedures implemented, and cessation plan written

## 5 GOVERNANCE STRATEGIES TO MAINTAIN EFFICIENT, EFFECTIVE AND ETHICAL CARE

- Review whole‐of‐hospital policy, procedures and guidelines, risk management systems, clinical and support staff training (Figure 1)
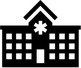

- Separate wards and staff with health‐care workers skilled in managing cognitive impairment challenges
- Enable hospital avoidance strategies if safe to do so
- Enable hospital stay to include recovery, restorative care and rehabilitation

## Conflicts of interest

No conflicts of interest declared.

## Appendix 1

### Cognitive Impairment and COVID‐19, Hospital Care Guidance Committee Membership

Dr Melinda Martin‐Khan, Chair, Centre for Health Services Research, The University of Queensland, Australia ; Dr Alison Argo, Statewide Dementia Clinical Network; A/Prof Kasia Bail, University of Canberra; A/Prof Gideon Caplan, Prince of Wales Hospital; Ms Denise Craig, Cairns and Hinterland Hospital and Health Service, James Cook University School of Medicine and Dentistry; Anne Cumming, Australian Commission on Safety and Quality in Health Care; Stephanie Ellis, NHMRC National Institute for Dementia Research; Leon Flicker, Western Australian Centre for Health and Ageing, Medical School, University of Western Australia; Dr Amanda Fox, Centre for Healthcare Transformation, Faculty of Health, Queensland University of Technology, Australia; Dennis Frost, Dementia Australia Advisory Committee; Dr Jennifer Galstuch‐Leon, Specialist Mental Health Intellectual Disability Service (SMHIDS), West Moreton Health; Frederick Graham, Princess Alexandra Hospital; Annemarie Hosie, The University of Notre Dame, School of Nursing Sydney; Juanita Hughes, Dementia Australia Advisory Committee; Leanne Jack, School of Nursing Queensland University of Technology, Australia; David Lie, Older Adult Mental Health, Metro South Addiction & Mental Health; A/Prof. R. J. Soares Magalhães, School of Veterinary Science, Children's Health Research Centre, The University of Queensland; Elizabeth Miller, Health Consumers Qld/QH Collaborative Member; Glenys Petrie, Metro South Health; Dr. Ranjeev Chrysanth Pulle, Qld Division of ANZSGM 2017‐21; John Quinn, Metro South Health; Bobby Redman, Health Services Consumer/Advocate; Dr Linda Schnitker, School of Nursing, Queensland University of Technology; Prof. Christine Stirling, University of Tasmania; Adjunct Prof. Eddy Strivens, Cairns and Hinterland Hospital and Health Service, James Cook University School of Medicine and Dentistry; Prof. Mark Yates, Deakin University, Ballarat Health Services, Ballarat Innovation and Research Collaboration For Health.

