# Supplementary material for: Interim guidance for health‐care professionals and administrators providing hospital care to adult patients with cognitive impairment, in the context of COVID‐19 pandemic
Source: Australas J Ageing. 2020 Oct 13;39(3):283–6. doi: 10.1111/ajag.12831 (PMC7675584; doi:10.1111/ajag.12831)
Supplement: Supplementary file 1 [file AJAG-39-283-s001.pdf]

Patients with cognitive impairment in hospital

during the COVID-19 pandemic

THE UNIVERSITY OF QUEENSLAND
AUSTRALIA
CREATE CHANGE

Interim guidance for health care professionals and administrators providing hospital care to adult patients with cognitive impairment, in the context of COVID-19 pandemic. More information at https://chsr.centre.uq.edu.au/interim-guidance-care-adult-patients-cognitive-impairment-requiring-hospital-care-during-covid-19-pandemic-australia.

Cognitive impairment may increase during COVID-19:

- **COVID-19 can cause delirium**
- **Admissions may increase for patients with dementia or intellectual disability** due to COVID-19 spatial isolation and reduced community resources
- **People with any kind of cognitive impairment are at higher risk of complications and distress,** e.g. adverse events, long length of stay, behavioural and psychological symptoms and death
- **Higher risk warrants increased preventative strategies to reduce the risk of harm**

People with cognitive impairment may require innovative approaches to care because of:

- **Inconsistent historians, comprehension of care requirements, remembering/following instructions**
- **Challenges in maintaining infection control principles** (e.g. keeping mask on) due to the person experiencing anxiety, restlessness, breathlessness, exit-seeking behaviours/wandering, fear, agitation, or aggression
- **Limited access to their usual care partner/advocate** (e.g. due to COVID-19 control measures or illness)
- **Fear of people wearing personal protective equipment (PPE)** which can be frightening and unfamiliar

Clinical strategies to maintain efficient, effective and ethical care:

- **Identify contributing factors to delirium and factors that are treatable**
  - Manage hypoxia, pain, infection, dehydration, constipation, hunger, strange environments
  - Reduce polypharmacy and tethers where possible (IVC, IDC, bed rails)

- **Normalise infection control practices**
  - Use regular calm reorienting conversations, maintain calm demeanor, prioritise dignity and respect
  - Provide sample packs of PPE to enable familiarization for people with cognitive impairment
  - Consider humanisation of health professionals by placing large print name labels and photos on health professionals wearing PPE
  - Consider best environment for individuals based on their acceptance of PPE
  - Provide education on PPE and infection control to care partner/advocate who will be present in hospital

- **Orient people with cognitive impairment using biopsychosocial reinforcement**
  - Welcome care partner/advocate to stay with people with cognitive impairment
  - Document the 'Top 5' strategies that were requested by the person (or care partner/advocate) for help with their care in their medical record
  - Place items in view (family photos, music, phone, personal items)
  - Encourage activity (life story book/app, puzzles, fidget boards, towel folding, tool-box)
  - Use human solutions (hearing and visual aids, music, pictures, tv, video)
  - Support time orientation: day/night lighting; bedside clock/calendar; assist with meals
  - Promote the use of staff familiar to the patient; social and mobilizing time
  - Write down information and instructions for patients, use visible whiteboard

- **Discuss and document goals of care**
  - Identify the lawful decision-maker if substitute decision making is occurring
  - Support shared decision making, informed consent, and advance care planning
  - Plan comprehensive care based on goals of care and in line with values and preferences, ensure regular communication
  - Focus on reablement, palliative care or end of life care as relevant

- **Respond to any behavioural deterioration (breach of infection control, aggressive behavior) (Fig. 1 applies to all people with cognitive impairment with or without COVID-19)**
  - Implement non-pharmacological strategies (as above)
  - Medications should be avoided and used only in extreme circumstances in a timely manner with consent policies and procedures implemented, and cessation plan written

Governance strategies to maintain efficient, effective and ethical care:

- **Review whole-of-hospital policy, procedures and guidelines, risk management systems, clinical and support staff training (Fig. 1 applies to all people with cognitive impairment with or without COVID-19)**
- **Separate wards and staff with healthcare workers skilled in managing cognitive impairment challenges**
- **Enable hospital avoidance strategies if safe to do so**
- **Enable hospital stay to include recovery, restorative care and rehabilitation**

Figure 1: Flowchart of COVID-19 clinical strategies for people with cognitive impairment

SCREEN TO IDENTIFY COGNITIVE IMPAIRMENT (including delirium)

- Use your facility (low contact) cognitive/delirium screen (e.g. 4AT, CAM)
- Determine any dementia, delirium, brain injury, intellectual disability (identify established diagnosis, history, concerns)
- Ensure all health care professionals are aware of the person's cognitive impairment

Is there agitation, aggression or exit seeking?
Do symptoms undermine infection control?

YES → Single Room COVID-19 ward +/- extra nurse resourcing

NO → COVID-19 ward/bed +/- extra nurse resourcing

PREVENTATIVE STRATEGIES

People with Cognitive Impairment

PAIN: prioritise pain as a trigger. If pain is suspected, perform an analgesic trial and evaluate using patient's self-report and observational pain tools.
CLINICAL PROVOCATIONS: monitor using ***PITCHED*** *– **P**ain **I**nfection **T**hirst **C**onstipation **H**unger **E**nvironment **D**rugs*
SUNFLOWER: contact family, complete and display positive life story info on chart to facilitate person-centred communication
TOP 5: contact family and document in progress notes "Tell me 5 things I need to know about caring for (X)?
NORMALISE: attempt to normalise PPE context with regular calm orienting conversations. Wear name and photo on PPE
HEALTH LITERACY: where appropriate, individualise education about the situation
SOCIAL: plan for staff to spend periods of social time with patient, spaced out regularly across a shift (PPE limited)
ACTIVITY: encourage recreational activity. Ask families to provide an activity box (life story book & resources)
FAMILIARITY: use iPad to play video messages that family/friends have been encouraged to prerecord (1-2 minute message)

BEHAVIOURAL PLAN for acute, severe behaviour with immediate risk of harm

When symptom severity threatens safety for person or others and has not reduced with preventative strategies

Prescribe (in advance) PRN analgesia for possible pain related behaviour
Non-pharmacological strategies should be used first and medications used as the last option.

RESPONSE TO ACUTE, SEVERE BEHAVIOUR

Request extra assistance (if required)

Administer analgesia first, if any suspected reasons for pain (patients often unable to self-report)
Psychotropic medications may paradoxically worsen symptoms in some patients. (Use existing prescribed medications; Note cautions for Lewy Body Disease and Parkinson's Disease - Quetiapine has the lowest risk of causing significant extrapyramidal side effects in patients with Lewy body dementia and Parkinson's disease dementia).

Sedating agents carry considerable risk in acutely unwell patients, have limited evidence base and should be avoided
Studies have shown that older adults with dementia who take antipsychotics have an increased chance of death during treatment. Write cessation plan. Explain use and risks to person with cognitive impairment if feasible, and to care partner/advocate.

Some examples of medication doses (based on COVID-19 guidance from the British Geriatric Society and UK Royal College of Psychiatry, and using existing guidelines for delirium and dementia by the Royal Australian and New Zealand College of Psychiatrists, NPS Medicinewise, and the Australian Clinical Practice Guidelines) have been provided to highlight the importance of starting low and going slow when using medication in situations of acute deterioration.

1st line: **Haloperidol (IM or O) or risperidone 0.25 to 0.5 mg every 4 hours** up to a maximum of 2mg over 24 hours.
2nd line: If ineffective then small doses such as olanzapine (2.5 mg prn four hourly up to a maximum of 10mg over 24 hours) or quetiapine (12.5 mg to 25 mg prn four hourly up to a maximum of 100mg over 24 hours).

Benzodiazepines e.g. midazolam and lorazepam are best avoided considering respiratory depression in patients with COVID-19. If used, senior staff should be consulted and clear goals of care regarding ventilation and palliation established.

Adhere to organisation policies and legislative requirements National Guidelines:
Clinical practice guidelines and principles of care for people with dementia, Delirium Clinical Care Standard

CRICOS Provider Number 00025B
